# Novel application of modified coronally advanced flap with a vertical incision for the management of multiple gingival recessions: A case report

**DOI:** 10.34172/japid.026.3898

**Published:** 2026-05-11

**Authors:** Shivaprasad Shivaprasad, Vaishnavi Pandey

**Affiliations:** ^1^Manipal College of Dental Sciences, Manipal, India; ^2^Manipal Academy of Higher Education, Manipal, India

**Keywords:** Aesthetic, Case report, Dentin hypersensitivity, Gingival recessions, Periodontics, Plastic surgery

## Abstract

Gingival recession presents both aesthetic and functional challenges, often requiring surgical correction to achieve root coverage and tissue stability. This case report describes a novel modification of the coronally advanced flap (CAF) technique that combines a single vertical incision with Zucchelli’s envelope design for managing multiple adjacent Miller’s Class I recessions. The approach aims to enhance vascularity, flap mobility, and aesthetic integration while minimizing surgical trauma. With this rationale, the case report describes a 45-year-old male with root exposure and dentin hypersensitivity in teeth #13 to #16 due to traumatic brushing. Clinical examinations revealed Miller’s Class I recession, probing depths≤2 mm, and a thick gingival phenotype. A modified CAF with a split-full-split thickness design and a single vertical incision was performed following de-epithelialization of the interdental papillae. The flap was coronally advanced and secured without tension. Healing was uneventful, with partial (70%) root coverage at 1 month and complete (100%) root coverage achieved by 3 months. This was maintained with an average gain of 2 mm in keratinized tissue and harmonious color blending at the 12-month follow-up. This technique provides a predictable and aesthetically refined solution for multiple adjacent recessions, offering a promising alternative to conventional CAF procedures.

## Graphical Abstract

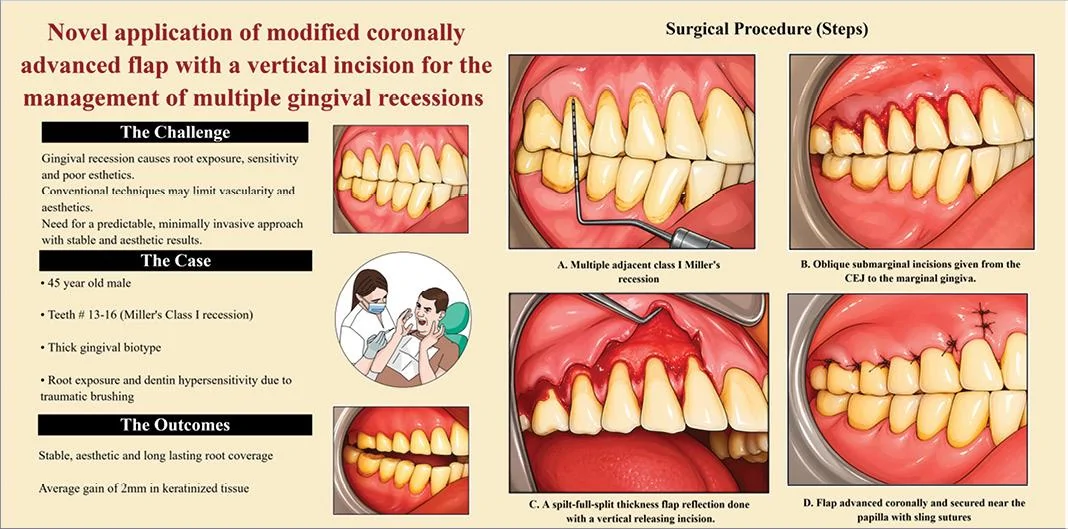

## Introduction

 The apical shift of the marginal gingiva from the cementoenamel junction (CEJ), exposing the tooth root, is referred to as gingival recession.^1^ Several factors can contribute to this mucogingival defect, including faulty toothbrushing techniques, plaque-induced periodontitis, tooth malpositioning, high frenal attachment, and ongoing orthodontic therapy.^2^ From the patient’s perspective, the most common concerns related to gingival recession are dissatisfaction with aesthetic and dentin hypersensitivity.^3^ Among the primary goals of root coverage procedures, enhancing the patient’s aesthetic outcome should be a top priority.^1^

 Surgical techniques for managing gingival recession aim to achieve full 100% coverage of the exposed roots with the attached keratinized gingiva adjacent to the recession defects.^4^ In the case of multiple recession defects, the coronally advanced flap (CAF) is better suited when there is a sufficient amount of keratinized tissue apical to the defect. This technique is considered reliable in periodontal plastic surgery, offering optimal root coverage, good color blending, and satisfactory restoration of gingival morphology. Over the years, various modifications of the coronally advanced flap have been proposed to enhance predictability and aesthetic outcomes in multiple recession defects. Among them, Zucchelli and De Sanctis (2000) introduced an envelope design without vertical releasing incisions, which preserves the lateral blood supply and allows simultaneous treatment of multiple adjacent recessions.^5^ Several clinical trials have reported mean root coverage ranging from 90% to 97% with this approach, along with superior color match and reduced postoperative morbidity compared with traditional CAF techniques. To improve outcomes, the CAF technique has been modified to provide maximum root coverage in cases involving multiple adjacent gingival recessions, particularly Miller’s Class I and Class II defects, within a single surgical session.^6^ One such approach is the split-full-split envelope flap technique with coronal mobilization, which results in up to 97% root coverage while maintaining optimal soft tissue thickness.^7^ The absence of vertical releasing incisions in the envelope flap design offers significant benefits in terms of both vascular supply and aesthetics. This design avoids disruption of the lateral blood supply and prevents the formation of unaesthetic visible white scars.^8^

 Given its favorable characteristics, such as harmonious tissue blending and stable flap positioning without disrupting the blood supply, the modified coronally positioned flap with an envelope technique represents a valuable approach for treating multiple gingival recession defects. However, despite its predictable outcomes, there remains limited clinical documentation of this technique and long-term follow-up data. These reviews emphasize the relative lack of high-quality data specifically addressing posterior sites and technical refinements (such as combining an envelope design with a single vertical incision), supporting the need for case-level documentation.^9,10^

 Hence, this case report aims to demonstrate the clinical efficacy, aesthetic integration, and stability of the modified coronally advanced flap using Zucchelli’s envelope technique in the management of multiple adjacent gingival recessions. This case report explores its effectiveness and reliability in addressing the needs of patients with high aesthetic expectations. It has been prepared and reported in accordance with the CARE (CAse REport) guidelines.

## Case Report

 A 45-year-old male patient visited the Department of Periodontics at the Manipal College of Dental Sciences with the primary concern of unsatisfactory aesthetics due to gingival recession in the upper right quadrant, with associated dentin hypersensitivity. The patient had no relevant familial history, demonstrated good plaque control, and had no history of systemic conditions or deleterious habits that could compromise periodontal wound healing. During the consultation, it was noted that he used a horizontal scrub technique while brushing, which was identified as a contributing factor to the recession.

 Clinical evaluation revealed Miller’s Class I gingival recession affecting teeth #13, #14, #5, and #16. The recorded recession depths were 3 and 2 mm, with corresponding widths of 4 and 3 mm, respectively. Overall, the periodontium appeared healthy, apart from mild marginal inflammation. The gingival phenotype was found to be thick. High-resolution preoperative photographs were captured via a DSLR camera with standardized positioning (Figures 1 and 2). Written informed consent was obtained from the patient for both treatment and the publication of anonymized clinical data and images.

**Figure 1:**
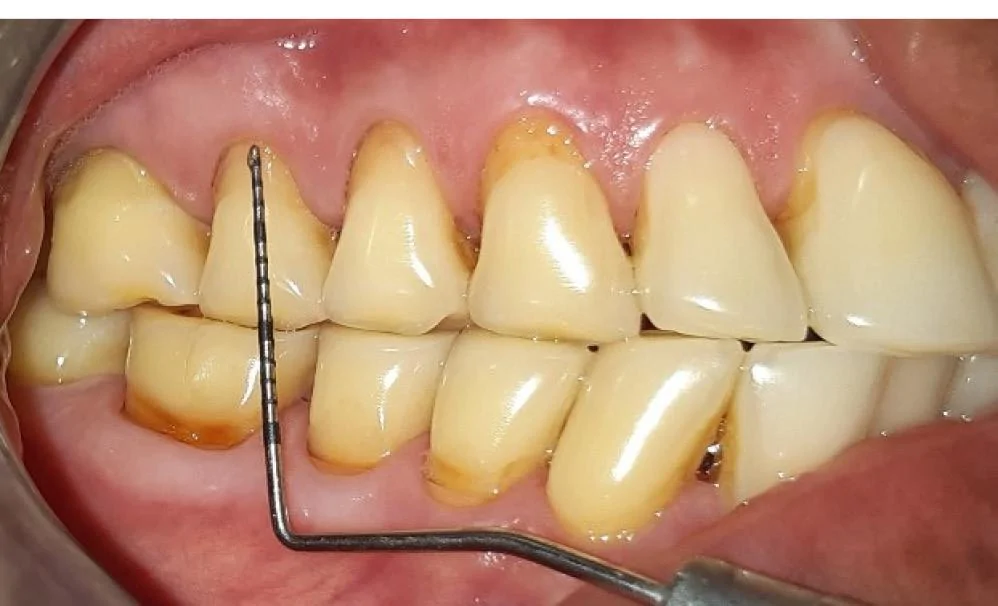
Canine, premolars, and the first molar showing gingival recession defects from the CEJ to the gingival margin

**Figure 2:**
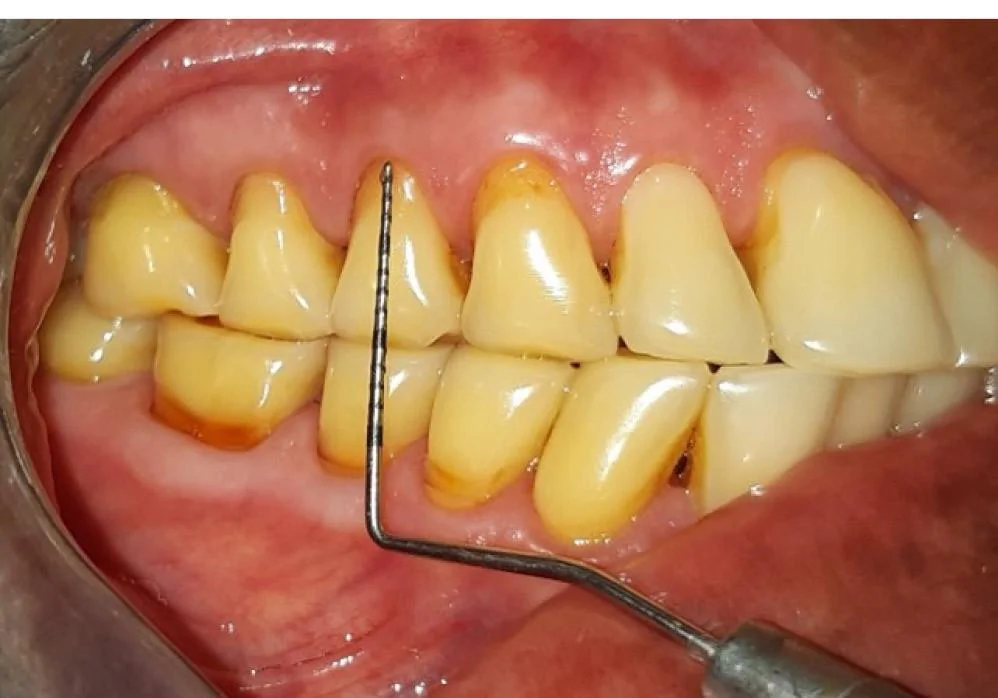
UNC #15 probe was used to measure the recession depth from the CEJ to the gingival margin

 Based on the clinical presentation and favorable local and systemic factors, a modified coronally advanced flap with vertical incision was selected as the treatment approach to achieve optimal root coverage.

###  Presurgical Procedure

 Following patient evaluation, oral prophylaxis was performed, which included professional tooth cleaning, scaling, and polishing with a prophylactic polishing paste. To reduce further trauma to the gingival margin, a roll-type brushing technique was implemented. A preoperative 0.12% chlorhexidine rinse was advised twice daily for one week before surgery. Surgical intervention was postponed until the patient demonstrated satisfactory supragingival plaque control.

###  Surgical Procedure

 The surgery involved a novel technique in which the original coronally positioned flap was modified for multiple recession defects. The modified coronally positioned flap procedure was performed under local anesthesia, with local infiltration anesthesia administered in the vestibular region of the surgical site.

 A key characteristic of this technique is the presence of a single vertical releasing incision and the raising of a flap with variable partial-thickness areas, combining both split- and full-thickness areas with coronal repositioning. The incisions in the papillary area connecting the CEJ of one tooth to the gingival margin of the adjacent tooth were oblique in nature. A similar pattern was followed for the horizontal incisions (Figure 3).

**Figure 3:**
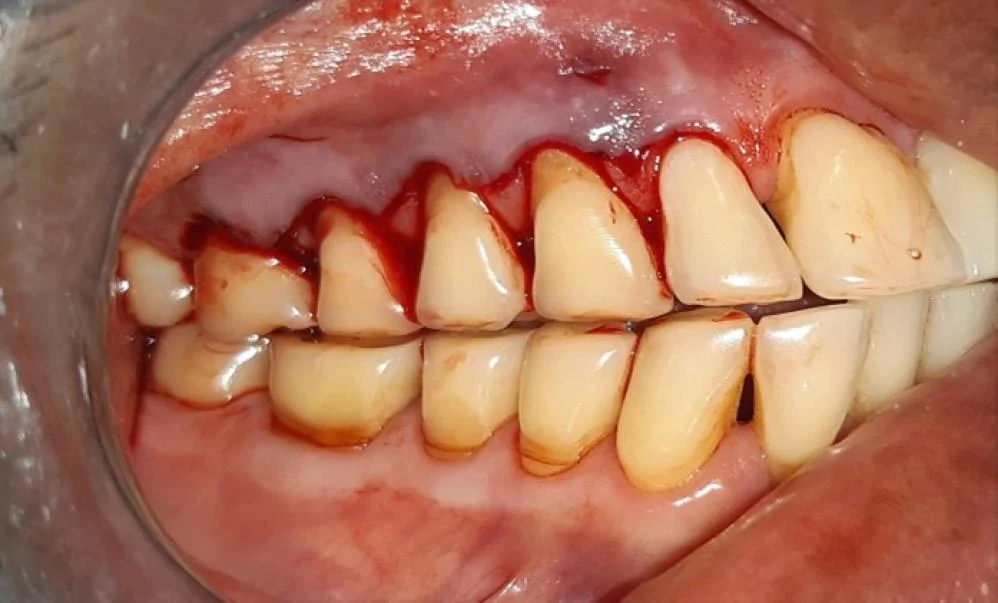
Oblique submarginal incisions given from the CEJ to the marginal gingiva, along with the sulcular incisions

 Initially, a partial-thickness flap was carefully elevated to the extent of the exposed root surfaces, minimizing trauma to the underlying structures (Figure 4). Similarly, a full-thickness flap was raised to ensure adequate exposure and access to the underlying bone and root surfaces. As the elevation progressed across the mucogingival junction, the flap again transitioned into a split-thickness configuration (Figure 5). This step is critical for achieving sufficient flap mobility, which is essential for tension-free coronal repositioning.

**Figure 4:**
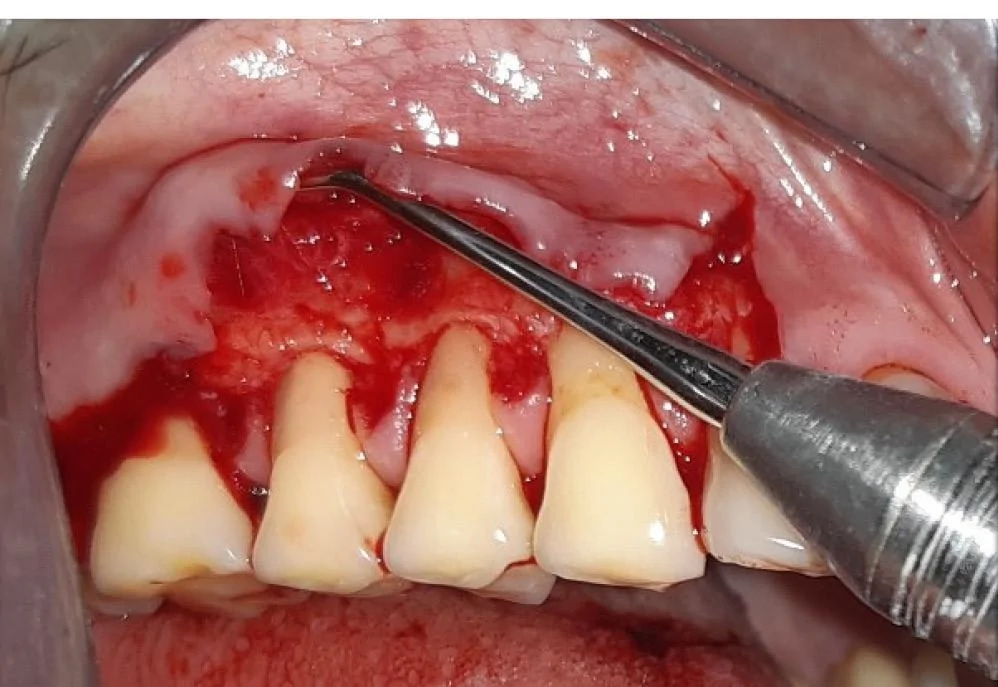
A split-thickness flap raised at interdental areas

**Figure 5:**
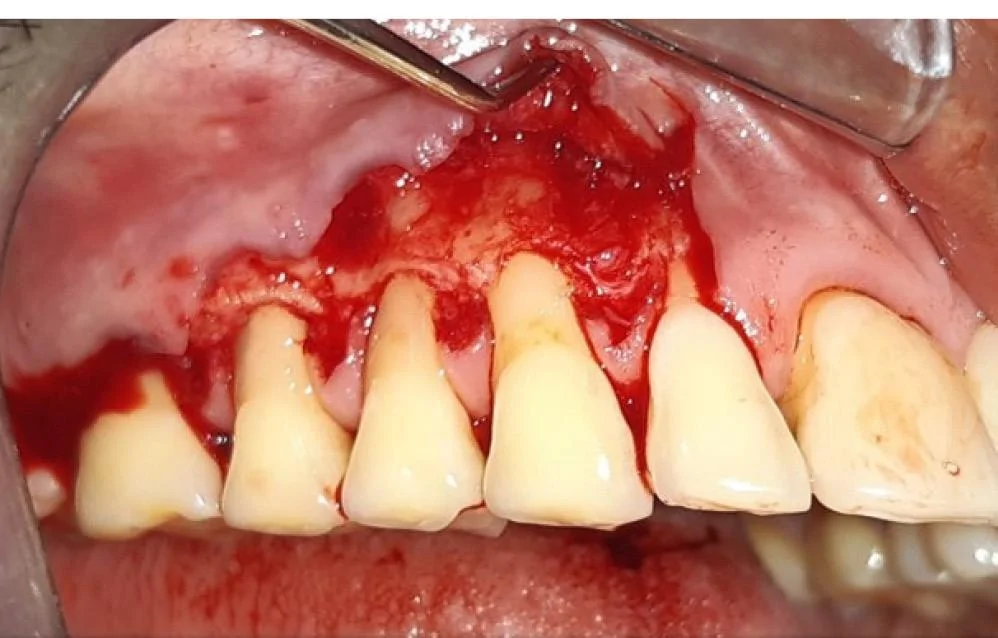
A full-thickness flap was raised beyond the gingival margins, a split-thickness flap was raised beyond the mucogingival junction, and the root planing was done

 To prepare the recipient site, the anatomical interdental papillae were meticulously de-epithelialized with a microsurgical ophthalmic blade using surgical loupes at × 3.5 magnification (Figure 6). This was done to reveal the underlying connective tissue and remove any epithelial remnants that could impede its integration and wound healing.

**Figure 6:**
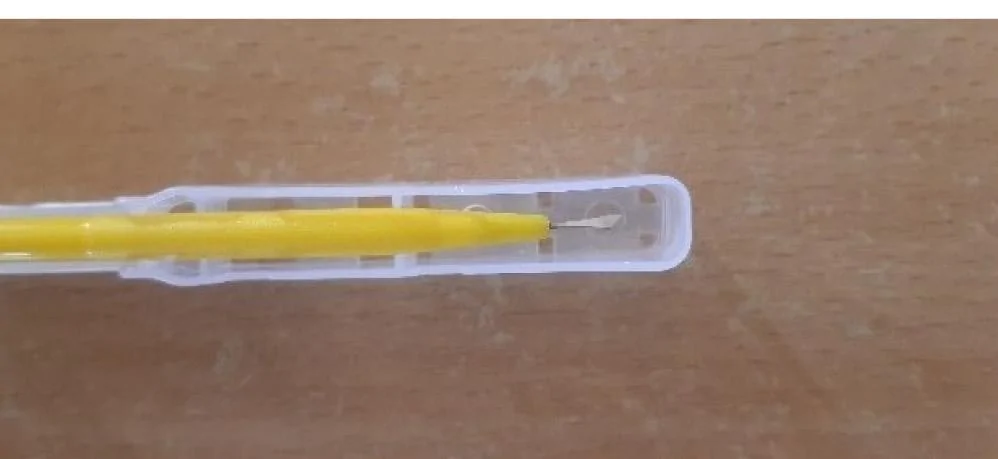
A microsurgical ophthalmic blade was used to de-epithelialize the papilla

 Upon complete reflection of the flap, the root surfaces were thoroughly assessed for the presence of any remaining calculus deposits. Meticulous scaling and root planing were performed to ensure a clean, biologically compatible root surface conducive to soft tissue reattachment.

 During the coronal positioning of the flap, the surgically created papillae were rotated and precisely positioned over the connective tissue beds of the previously de-epithelialized interdental papillae. This step was executed with careful attention to ensure optimal adaptation and stability. The entire flap was then secured in position with sling sutures (Figure 7), which provided tension-free stabilization and promoted close contact with the underlying tissues.

**Figure 7:**
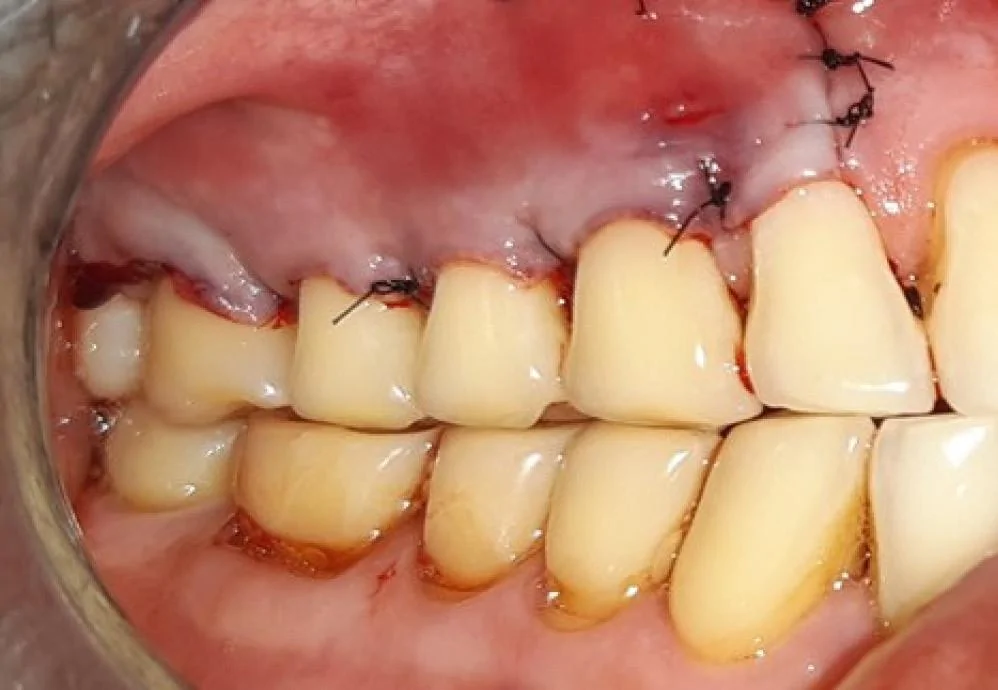
Sling sutures were used to stabilize the coronally advanced flap, and simple interrupted sutures were used for the vertical releasing incisions

 Finally, a periodontal pack was applied over the surgical site to protect the area during the initial healing phase (Figure 8). This dressing serves as a physical barrier against mechanical trauma and microbial contamination, assisting in an undisturbed healing environment and favorable postoperative outcomes.

**Figure 8:**
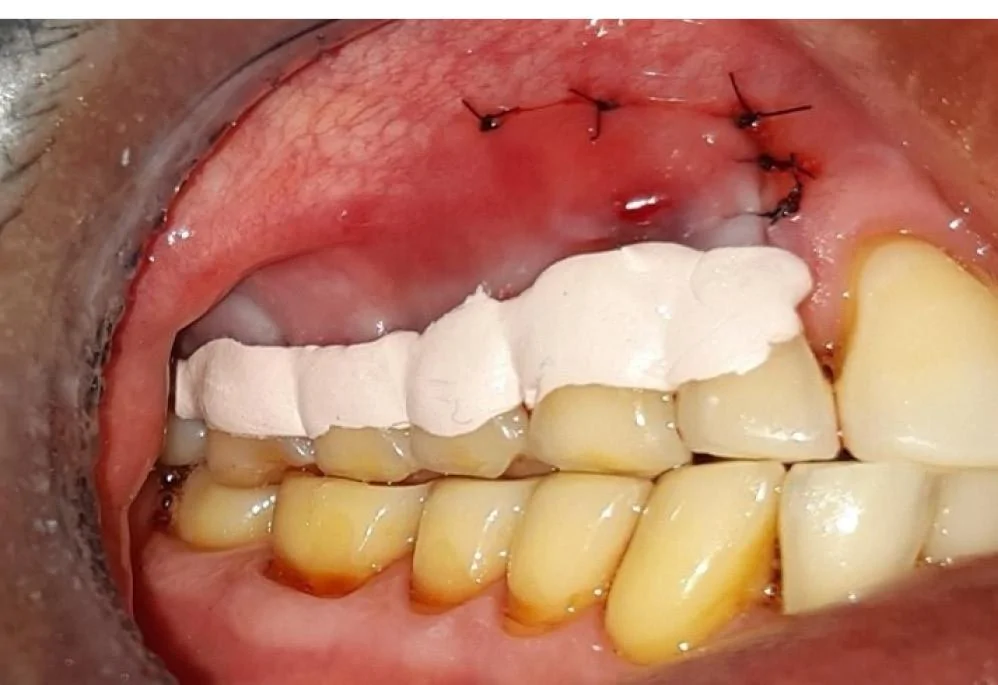
Periodontal pack placed

###  Postoperative Instructions

 Postoperatively, the patient was prescribed diclofenac (50 mg tid) and 0.12% chlorhexidine rinse twice daily for 2 weeks. The sutures and periodontal dressing were removed after 2 weeks. Follow-up visits were scheduled at 1, 3, 6, and 12 months, during which recession depth, keratinized tissue width, and patient-reported outcomes such as dentin hypersensitivity and aesthetic satisfaction were recorded.

Tables 1 and 2 present the detailed surgical protocol and patient timeline for enhanced clinical reproducibility.

**Table 1:** Surgical and perioperative details for clinical reproducibility, including a comprehensive summary of intraoperative parameters, flap design, instrumentation, and perioperative management, to increase the reproducibility of the modified Zucchelli’s envelope technique with a single vertical releasing incision

**Step**	**Detail**	**Description/Instruments Used**
Flap design	Split-full-split thickness envelope flap	Partial-thickness elevation over roots, full-thickness in the mid-section, and split-thickness beyond the mucogingival junction for coronal mobility
Incision pattern	Single vertical releasing incision	Placed mesial to tooth 13 to enhance access without compromising vascularity
Papilla management	De-epithelialization	Microsurgical ophthalmic blade under × 3.5 magnification loupes
Root surface preparation	Scaling and root planing	Gracey curettes (Hu-Friedy), followed by saline irrigation
Flap advancement and fixation	Coronal repositioning and sling sutures	5-0 resorbable sutures (Vicryl Rapide^TM^) ensuring tension-free closure
Periodontal dressing	Coe-Pak^TM^	Applied over the surgical site for 7 days
Postoperative care	Analgesic and antiseptic regimen	Tab. Diclofenac 50 mg TID × 3 days; 0.12% chlorhexidine rinse BID for 2 weeks
Oral hygiene reinforcement	Modified Stillman brushing technique	Reinforced at each follow-up visit

**Table 2:** Chronological representation of preoperative, intraoperative, and postoperative milestones, outlining clinical progression, healing events, and patient-reported outcomes during the 12-month follow-up

**Time point**	**Clinical step**	**Observation/Outcome**
Day 0	Baseline evaluation	3-mm recession (teeth 13–16), probing depths ≤ 2 mm
Day 7	Surgery performed	Modified CAF with a single vertical incision was completed
Week 1	Initial review	Stable flap, no inflammation
Week 2	Suture & pack removal	Uneventful healing
Month 1	Early outcome	Partial root coverage (70%), healthy marginal contour
Month 3	Intermediate outcome	100% root coverage achieved; average 2mm gain in keratinized tissue
Month 6	Short-term follow-up	Stable results, no dentin hypersensitivity
Month 12	Long-term follow-up	Maintained root coverage, excellent color match, no recurrence

 At baseline, the mean gingival recession depth was 3.0 ± 0.2 mm across teeth #13–16, with probing depths ≤ 2 mm and an average of 2 mm of keratinized tissue apical to the recession defects. The surgical procedure was well tolerated, and healing progressed uneventfully.

 At the 1- and 2-week intervals, the flap margins remained stable, with no signs of inflammation, necrosis, or marginal opening. By 1-month, partial root coverage (70%) and satisfactory tissue adaptation were noted. At 3 months, complete (100%) root coverage was achieved, with a mean gain of 2 mm in keratinized tissue width and uniform color integration.

 At the 6- and 12-month evaluations, the outcomes remained stable, maintaining full root coverage and probing depths at ≤ 2 mm, with no signs of marginal relapse, scarring, or color mismatch (Figure 9). The patient reported sustained relief from dentin hypersensitivity and high satisfaction with the aesthetic outcome.

**Figure 9:**
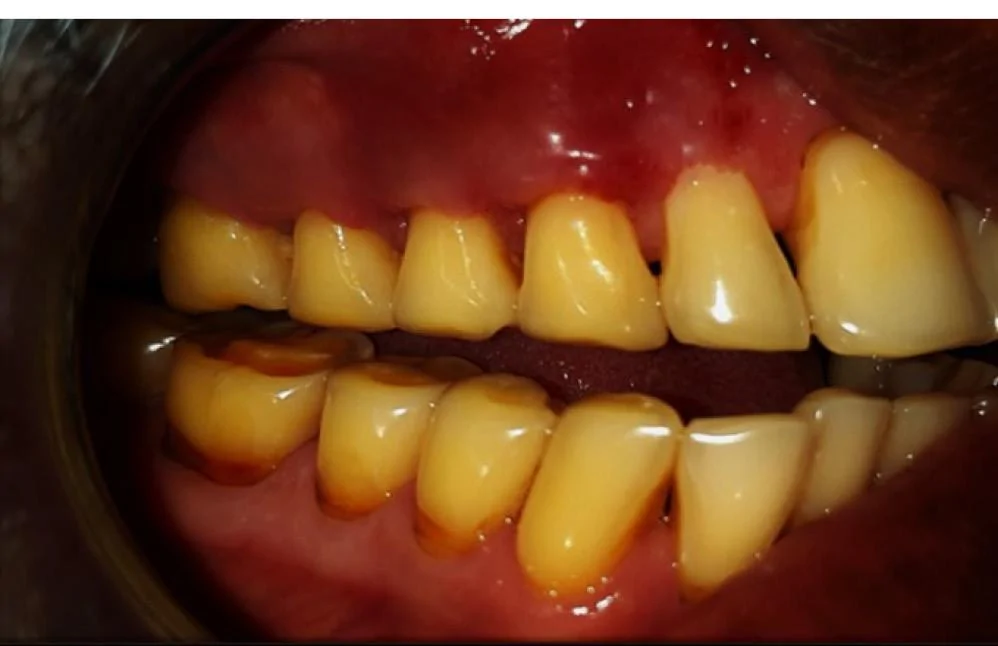
Complete root coverage was achieved with stable results after 6 months

 Overall, the modified CAF technique yielded predictable quantitative improvements and stable periodontal architecture up to 12 months postoperatively.

## Discussion

 The patient’s aesthetic demands may be a chief indication for performing root coverage surgical procedures. Therefore, the primary goal here was to restore and achieve complete root coverage up to the CEJ to improve the aesthetic appearance of the teeth. Patient-centered outcomes, such as a reduction in dentin hypersensitivity and improvements in smile confidence, also play a pivotal role in assessing the success of root coverage procedures, especially when aesthetics is the primary concern.^11^

 To facilitate adequate root coverage, a coronally advanced flap approach is a known therapeutic option that displaces soft tissue coronally to cover receded root surfaces.^12^ The present modification of the coronally advanced flap allows coronal displacement by eliminating muscle insertions to remove tension and provide passive displacement of the flap until the CEJ without any muscle pull.^7^ This adaptation minimizes postoperative discomfort and improves vascular integrity, which are critical determinants of predictable healing.

 The unique clinical contribution of the present case lies in integrating Zucchelli’s envelope design with a single vertical releasing incision, thereby enhancing flap mobility and preserving vascular integrity. Unlike traditional bilateral incisions, this hybrid design minimizes tissue trauma while maintaining the visibility and flexibility necessary for precise coronal advancement. This subtle yet effective modification provides an operative advantage in managing multiple adjacent recessions, especially in posterior aesthetic zones that are often underrepresented in the literature. Compared with alternative techniques such as the laterally positioned flap, connective tissue graft, or tunnel approach, the modified coronally advanced flap offers the advantages of reduced donor-site morbidity, minimal scar formation, and better color blending with adjacent tissues. Furthermore, it is less technique-sensitive and ensures predictable flap stability even at posterior sites.

 The coronally advanced flap (CAF) technique was initially established as a reliable, evidence-based approach for achieving root coverage in cases of isolated gingival recession. However, gingival recessions more commonly affect multiple adjacent teeth. A systematic review by Hofmänner et al.^13^ demonstrated that complete root coverage can be successfully achieved via a modified CAF technique in the treatment of Miller Class I and II multiple recession defects, with the results remaining stable over a 5-year follow-up period.^13^ In a systematic review and meta-analysis by Graziani et al.,^14^ a modified coronally positioned flap showed full root coverage, with the highest level in managing multiple gingival recessions.^14^

 In the present case, complete coverage of the 3-mm gingival recession was achieved, with a noticeable gain in keratinized tissue width and harmonious tissue contour. The patient expressed high satisfaction with the aesthetic outcome and resolution of dentin hypersensitivity during follow-up. In accordance with these studies, this case report demonstrated the clinical outcome of a modified coronally advanced flap in treating multiple contiguous recession defects in patients with high aesthetic demands. This technique offers certain advantages over conventional procedures, such as the split-full-split thickness flap approach, which guarantees precise coronal positioning of the flap with stable anchorage and rich vascularization to the surgical interdental papillae.

 In the present case, a single vertical releasing incision was used, as a systematic review reported that coronally advanced flaps (CAFs) with vertical releasing incisions provide sufficient flap mobility, enhanced visibility, and facilitate coronal repositioning of the marginal gingiva, thereby improving root coverage.^15^ The 12-month postoperative evaluation confirmed the stability of the marginal tissue level with no recurrence or scarring, indicating the long-term reliability of the modified CAF approach.

 Treating mucogingival problems in molars can be unpredictable because of limited accessibility.^16^ However, in the present case, a modified coronally advanced flap procedure was performed on both the anterior and posterior teeth, including molars, to evaluate root coverage predictability. This flap technique provided satisfactory root coverage and optimal tissue color matching over 12 months, with no evidence of scarring or postoperative complications. Therefore, this case report demonstrated and supported the effectiveness of a modified coronally positioned flap in addressing multiple recession defects affecting adjacent teeth, resulting in enhanced root coverage and increased keratinized tissue.

 Despite favorable outcomes, this single-patient report has limitations, including a short follow-up duration and the absence of histological confirmation. Future randomized controlled trials with larger samples and long-term evaluations are warranted to validate these findings.

###  Patient Perspective 

 The patient reported marked improvements in comfort and confidence during smiling following surgery. Hypersensitivity previously experienced while brushing or consuming cold foods completely resolved within 2 weeks. The patient expressed high satisfaction with the aesthetic outcome and natural blending of the treated gingiva with adjacent tissues.

## Conclusion

 The modified coronally advanced flap integrating an envelope design with a single vertical incision may provide predictable root coverage and satisfactory aesthetic integration in multiple adjacent gingival recessions. Further controlled clinical studies are warranted to validate the reproducibility and long-term stability of this technique.

## Competing Interests

 The authors declare no competing interests related to this work.

## Consent to Publication

 Written informed consent was obtained from the patient for both the treatment procedure and the publication of anonymized clinical data and accompanying images in this journal.

## Data Availability

 All clinical and photographic data supporting the findings of this report are contained within the manuscript. All relevant data are included within the article.

## Ethical Approval

 Not applicable.
